# Editorial: Application of microbial technology in ecological remediation of mines

**DOI:** 10.3389/fmicb.2023.1136851

**Published:** 2023-02-13

**Authors:** Chunqiao Xiao, Chunli Zheng, Yanfei Zhang, Huan He, Sadia Ilyas

**Affiliations:** ^1^School of Environmental Ecology and Biological Engineering, Wuhan Institute of Technology, Wuhan, China; ^2^School of Energy and Environment, Inner Mongolia University of Science and Technology, Baotou, China; ^3^Tianjin Institute of Industrial Biotechnology, Chinese Academy of Sciences, Tianjin, China; ^4^School of Chemical Engineering and Technology, China University of Mining and Technology, Xuzhou, China; ^5^Department of Earth Resources and Environmental Engineering, Hanyang University, Seoul, Republic of Korea

**Keywords:** mining wastes, bioremediation, green technology, rehabilitation of mine sites, ecological remediation

Mining is the upstream activity to the mineral processing, energy exploitation, and metallurgical extraction of desired metals for the production of materials and energy, which is essential for human beings (Chen et al., 2020; Sonter et al., 2020). The continuous development of mining industries generates a large number of wastes which can cause the pollution of soil, groundwater, and air, and lead to serious hazards for various ecological systems (Balabanova et al., 2012; Heo et al., 2016; Jiang et al., 2021). Prolonged exposure to the contaminated mine sites is harmful to human health and could cause multiple retardations (Kang et al., 2016). The rehabilitation of mined land is therefore an imminent environmental issue. Compared to traditional physio-chemical remediation techniques, such as adsorption, ion exchange, precipitation, coagulation, solvent extraction, electrokinetic, etc., microbial treatment has gradually gained more attentions due to the advantages of low-cost and high environmentally friendliness (Jiang et al., 2011; Coban et al., 2022). Henceforth, mine remediation using microorganisms has been vastly studied over recent decade for remediation and ecological systems restoration at various mine sites.

The mechanistic approaches for remediation of mine sides varied greatly with variable microbial communities and contaminants (Matias et al., 2009), and many issues remain unresolved in field application. Given the knowledge gaps in the aforementioned contexts, a comprehensive understanding on an appropriate bioremediation approach is an absolute necessity. Therefore, the current Research Topic on the “*Application of microbial technology in ecological remediation of mines*” provides an overview to fulfill the knowledge gap in scenario of rehabilitation of effected mine sites. This Research Topic comprises five articles on various aspects albeit dealing with the ecological remediation of mines.

Liu et al. conducted a study on the bioremediation of waste drill cuttings-WDCs that comprise of rock cuttings (80%) and drilling muds (20%) using the bioaugmentation and phytoremediation techniques. They concluded that greenhouse incubated WDCs (over 120 days) with and without black locust plant (*Robinia pseudoacacia*) bacterial and fungal consortia in a combination mode could enhance the contaminant removal efficiency compared to natural attenuations.

Cockell et al. investigated the possibility of accomplishment of biological mining under extra-terrestrial gravitational conditions in ESA BioRock experiments. They demonstrated the potential use of microorganisms for mining activities and bio-industrial practices, in space locations, with non−1×*g* gravity. They have stated that same fundamentals are applicable to extra-terrestrial bioremediations and elemental recycling beyond the Earth.

The heavy metals polluted soil (from Xikuangshan in Lengshuijiang, Hunan Province, China) that particularly contains the highly toxic antimony and cadmium has been treated by Di et al.. The authors screened out a cadmium and antimony tolerant fungus namely, *Curvularia coatesiae* XK8 from a metallurgical waste (slag) that showed good potential as a biosorbent material to remediate the soil with a removal rate of 67.5%.

Ammoniacal-nitrogen contamination is an obstacle for sustainable development of rare earths industries. Hu et al. isolated *Pseudomonas mosselii* K17 (nitrifying-denitrifying, heterotrophic bacteria) from elution-deposited resources of rare earths located in Longnan county site of China. The strain, with an efficacy of about 95%, was capable to treat residual ammoniacal solution after leaching of rare earths.

Revegetation is an imperative indicator for restoration of ecosystems of mining area. In this context, Chang et al. observed the difference in key microbial clusters, their molecular ecological network, and their interactions under various vegetation restoration models and demonstrated the correlation of diverse vegetation restorations with microbial community diversities. Current work helps to effectively understand about natural restoration of ecosystems for ecologically damaged mining sites.

We hope that current collection of Research Topic on microbial remediation of the mining legacy will be useful for researchers of relevant domain.

## Author contributions

All authors listed have made a substantial, direct, and intellectual contribution to the work and approved it for publication.
